# Citizen science for monitoring the spatial and temporal dynamics of malaria vectors in relation to environmental risk factors in Ruhuha, Rwanda

**DOI:** 10.1186/s12936-021-03989-4

**Published:** 2021-12-03

**Authors:** Marilyn Milumbu Murindahabi, Arash Hoseni, L. C. Corné Vreugdenhil, Arnold J. H. van Vliet, Jackie Umupfasoni, Alphonse Mutabazi, Emmanuel Hakizimana, P. Marijn Poortvliet, Leon Mutesa, Willem Takken, Constantianus J. M. Koenraadt

**Affiliations:** 1 Laboratory of Entomology, Wageningen University & Research, Wageningen, The Netherlands; 2 College of Sciences and Technology, University of Rwanda, Kigali, Rwanda; 3 Laboratory of Geo-Information Science and Remote Sensing, Wageningen University & Research, Wageningen, The Netherlands; 4 Environmental Systems Analysis Group, Wageningen University & Research, Wageningen, The Netherlands; 5 Malaria and other Parasitic Diseases Division, Biomedical Center, Kigali, Rwanda; 6 Strategic Communication Group, Wageningen University & Research, Wageningen, The Netherlands; 7 College of Medicine and Health Sciences, University of Rwanda, Kigali, Rwanda

**Keywords:** Citizen science, Environmental risk factors, Malaria, Perceived mosquito nuisance, Rwanda

## Abstract

**Background:**

As part of malaria prevention and control efforts, the distribution and density of malaria mosquitoes requires continuous monitoring. Resources for long-term surveillance of malaria vectors, however, are often limited. The aim of the research was to evaluate the value of citizen science in providing insight into potential malaria vector hotspots and other malaria relevant information, and to determine predictors of malaria vector abundance in a region where routine mosquito monitoring has not been established to support vector surveillance.

**Methods:**

A 1-year citizen science programme for malaria mosquito surveillance was implemented in five villages of the Ruhuha sector in Bugesera district, Rwanda. In total, 112 volunteer citizens were enrolled and reported monthly data on mosquitoes collected in their peridomestic environment using handmade carbon-dioxide baited traps. Additionally, they reported mosquito nuisance experienced as well as the number of confirmed malaria cases in their household.

**Results:**

In total, 3793 female mosquitoes were collected, of which 10.8% were anophelines. For the entire period, 16% of the volunteers reported having at least one confirmed malaria case per month, but this varied by village and month. During the study year 66% of the households reported at least one malaria case. From a sector perspective, a higher mosquito and malaria vector abundance was observed in the two villages in the south of the study area. The findings revealed significant positive correlations among nuisance reported and confirmed malaria cases, and also between total number of Culicidae and confirmed malaria cases, but not between the numbers of the malaria vector *Anopheles gambiae* and malaria cases. At the sector level, of thirteen geographical risk factors considered for inclusion in multiple regression, distance to the river network and elevation played a role in explaining mosquito and malaria mosquito abundance.

**Conclusions:**

The study demonstrates that a citizen science approach can contribute to mosquito monitoring, and can help to identify areas that, in view of limited resources for control, are at higher risk of malaria.

## Background

Malaria is a major public health concern in Rwanda, and a leading cause of morbidity and mortality [1]. Despite the progress made in reducing the malaria burden over the last decades [2], the country experienced an upsurge of malaria since 2012, putting the entire population, including an estimated 443,000 pregnant women per year and 1.8 million children under 5 years, at risk of malaria [3]. This increase of malaria cases was especially observed in the East and the South provinces of the country. According to the Health Management Information System of the Malaria and Other Parasitic Disease Division (MOPDD-HMIS), these two provinces accounted for 79% of the disease burden [3].

Regardless of the malaria resurgence, Rwanda has made progress in vector monitoring by establishing 12 entomological sentinel sites that are involved in the surveillance of malaria vectors across the country [4]. This programme has given insight in the mosquito diversity, malaria vector and non-vector distribution and insecticide resistance status, as well as in entomological inoculation rates as a measure of transmission intensity [5]. Vector surveillance implies continuous monitoring of malaria mosquitoes [6]. This involves long-term sustained funding and trained entomologists, and also the physical infrastructure to accomplish such activities [6–8]. These activities receive external funding (70% from the Global Fund, President’s Malaria Initiative, and End Malaria Fund). Funds required to extend vector surveillance to regions other than the 12 sentinel sites are not available [1, 4]. Importantly, effective surveillance requires locality-specific information on the diversity, and the spatial and temporal distribution of malaria vectors to plan accordingly. This information helps to inform decision-making before outbreaks occur [9, 10]. Mostly, malaria prevention and control consists of early diagnosis and treatment as well as of vector control by the use of insecticide-treated bed nets (ITNs) and indoor residual spraying (IRS) [11]. The effectiveness of vector control is increased by accurate identification of the malaria vector population at the local level. Not identifying malaria vector hotspots can cause malaria prevention and control to fail [12, 13].

Several citizen science initiatives have demonstrated that citizens or community members can contribute to the monitoring of disease-carrying mosquitoes [14–21], but most of these have focused on settings outside of Africa. Innovation in malaria mosquito surveillance could therefore focus on how local communities and stakeholders, especially in a rural African context, can participate in citizen science with the aim of ensuring implementation and sustainability. Citizen science programmes represent a unique opportunity to involve the general public in the design, implementation, and evaluation of such vector surveillance programmes [18].

The main goals of the current study were to evaluate the value of a citizen science programme in providing insight into potential malaria vector hotspots and other malaria related information, and to determine predictors of malaria vector abundance in a region where routine mosquito monitoring has not been established. For this purpose, two research questions were formulated. First, what are the spatial and temporal dynamics in and correlations among (malaria) mosquito abundance, perceived mosquito nuisance and proportion of households reporting confirmed malaria cases in the study area? And second, what are the environmental drivers explaining the spatial and temporal distribution of the malaria vector *Anopheles gambiae* sensu lato (s.l.) and other mosquito species? The outcomes of this study will help to better understand malaria transmission dynamics in the study area based on citizen science data.

## Methods

### Study area

**Fig. 1:**
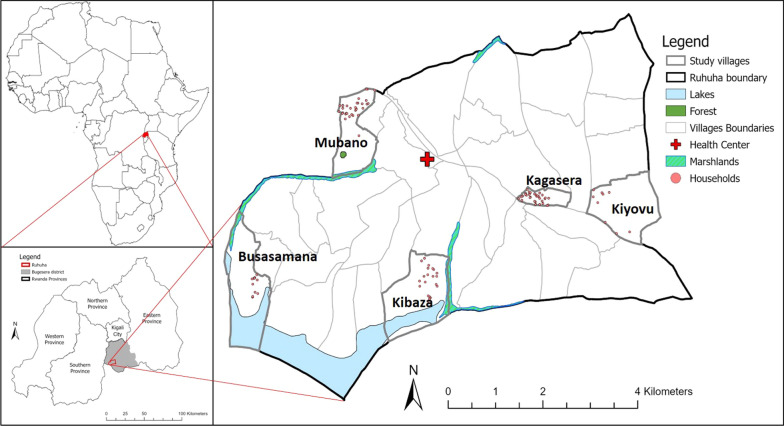
Map showing Ruhuha with the five selected villages where the citizen science programme was implemented (Busasamana, Kagasera, Kibaza, Kiyovu and Mubano). The pink dots represent the locations of the volunteers from where observations were reported

The study was conducted in five selected villages of the Ruhuha sector (Fig. 1) in the Bugesera district, of the Eastern province of Rwanda. The Ruhuha sector is composed of 35 villages grouped into five cells. The area covers 54 km^2^ and is located 42 km south of the capital Kigali [22]. The elevation varies from 1300 to 1573 m above sea level. It is surrounded by lowland marshes and water streams draining into the Akagera river system, and is separated from Burundi by Lake Cyohoha in the south [22]. The sector has an estimated population of 24,000 people, living in approximately 5000 households [23]. Ruhuha has a predominantly rural agricultural setting and is known as a malaria endemic area [22]. Ruhuha experiences two malaria transmission peaks associated with the rainy seasons observed generally from October to November and March to May [24].

### Recruitment of participants and distribution of materials and tools

**Fig. 2:**
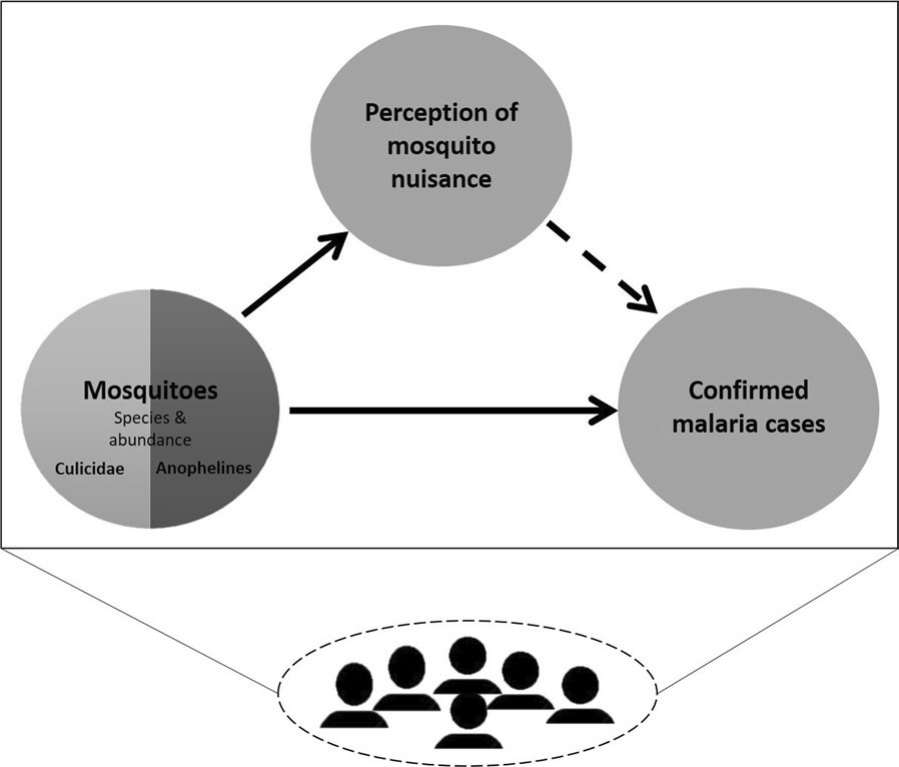
Diagram showing relations between variables and role of citizens in reporting data on these variables

Volunteer participants were recruited through workshops conducted 3 months prior to the implementation of the citizen science programme for malaria mosquito surveillance [25]. The list of volunteers consisted of the names of the participants and their contact details. Volunteers could indicate in which research activities they wanted to participate. This included filling forms with malaria relevant information and/or collecting mosquitoes. The citizen science programme kicked off with a launch event on 22 November 2018 in Ruhuha sector with participants from five selected villages of Ruhuha: Busasamana, Kagasera, Kibaza, Kiyovu, and Mubano (Fig. 1). Based on their preference, volunteers were requested to report every month the experienced mosquito nuisance and the number of malaria cases in their household, and to collect mosquitoes in their environment (Fig. 2).

To facilitate data collection, volunteers were grouped into groups of households (called *isibo*) close to each other. Each *isibo* consisted of a few households (3 to 10). A phone call was made by the researcher to the *isibo* leaders 1 or 2 days prior to data collection to remind them of their tasks and to collect the data properly. After four consecutive rounds of data collection, a dissemination workshop was organized to share the results from the data collected previously by the volunteers, and to motivate them to continue their active participation. Additionally, researchers could also check whether the correct procedures were followed in terms of filling the requested information on the paper forms or when and how to capture and conserve mosquitoes.

During the launch workshop, the data collection and reporting schemes of the observations were determined by the researchers in consultation with the volunteers. Paper forms, handmade traps, ingredients for production of carbon dioxide (sugar and yeast) for baiting the traps, batteries and torches were distributed during the launch workshop. These materials were used for the duration of the study except for some materials that needed to be replenished such as sugar, yeast, forms, and batteries. These were distributed monthly every last Friday by the *isibo* leader and distributed to the volunteers 3 days prior to the data collection during the monthly *isibo* meeting. Instructions on how to fill the forms and how to set up the traps as well as how to label the containers containing the mosquitoes and to store all the data collected, were given during the workshop. In addition, the *isibo* leaders, who represented the volunteers enrolled for the study in each selected village, were elected during the launch workshop as field data collectors. They were asked to assemble the data collected by the volunteers, and to submit these data to the researchers at Ruhuha health centre every last Friday of the month. *Isibo* leaders submitted the observations at the health centre three weeks prior to the next date of data collection. They also submitted a short report summarizing the data collected for the month and the challenges faced by the volunteers.

### Data collection

#### Mosquito nuisance and confirmed malaria cases

Collection of mosquitoes, and the reporting of nuisance experienced in the peridomestic area and other relevant malaria information took place from 28 November 2018 to 25 October 2019. The paper forms included two questions. One addressed the level of mosquito nuisance the participants perceived in their environment (indoors only, outdoors only and overall). The second question addressed whether participants had a confirmed malaria case in their household (diagnosed at the health centre using a blood sample) within the two weeks prior to the date of data collection. Next to these two questions, participants included the date, and personal information on the forms.

#### Mosquito collection and laboratory processing

In each household, mosquitoes were collected by placing two handmade traps that were baited with carbon dioxide and a torch (Murindahabi et al., submitted). The torch was hung approximately at 5 cm above the opening of the trap. One trap was placed indoors in the bedroom on the floor next to a human sleeping under a bed net, at the foot end of the bed. Another trap was placed outside of the house of the volunteers, preferably near the main entrance of the house and positioned on the ground against the wall. Carbon dioxide was provided by mixing 25 g brown sugar, 2 g of yeast and 250 mL of water at 9:00 a.m. In each trap, a gauze net was inserted to prevent mosquitoes from drowning in the sugar–yeast solution. The bottle was wrapped with a black scotch tape. The next day, mosquitoes were transferred to a petri dish, labelled with the collection date and time, and name of the volunteer. The observations were handed to the *isibo* leader who gathered all the forms and mosquitoes, and who sent them later to the researchers at Ruhuha health centre.

All mosquitoes were transported to the Mareba health centre and identified to species level using standard taxonomic keys [26, 27]. Mosquitoes were also scored as fed or unfed, then pooled per study site and stored in Eppendorf tubes with silica gel for transportation to the national laboratory in Kigali for further molecular analysis. The head and thorax of each individual female *An. gambiae* s.l. was used to determine the presence of circumsporozoite protein (CSP) of *Plasmodium falciparum* using enzyme-linked immunosorbent assay (ELISA) techniques [28]. The ELISA results were read visually [29]. Additionally, 10% of the total *An. gambiae* s.l. collected were used for sibling species identification by polymerase chain reaction (PCR) using the head and thorax of *An. gambiae* s.l. [30]. After DNA extraction, one microlitre of the DNA sample was used as the template for PCR amplification. Each amplified sample was run on a 2.5% agarose gel and visualized by a UV transilluminator.

#### Geographical data representing environmental drivers

To investigate whether publicly available data on geographic features within the sector could explain the observed patterns in mosquito abundance, variables were selected based on a review of relevant literature (Table 1). These environmental variables were mapped for the Ruhuha sector using publicly available data. From the Shuttle Radar Topography Mission (SRTM), the 30-m resolution Digital Elevation Model (DEM) was used to extract DEM derivates: Elevation, Slope, Aspect, hydrological Flow Accumulation, Topographical Position Index and Topographical Wetness Index. Based on the Flow Accumulation derived from the DEM, a river network was constructed and (Euclidean) distance to the river network calculated. Satellite imagery from Sentinel 2, level 2 A product, was used to derive 10-m resolution map layers for the Normalized Difference Vegetation Index (NDVI) and Normalized Difference Wetness Index (NDWI). Open water bodies were identified based on the NDWI and the Euclidean distance to these open water bodies was calculated. For the presence of marshland and the distance to it, data were used from the World Agroforestry Centre [31]. In addition, the population density of the area was collected from WorldPop [32] at a resolution of 3 arcseconds (approximately 100 m at the equator).

**Table 1:** Overview of the thirteen environmental variables selected for this study

Variable	Source	Spatial resolution	Temporal resolution	Authors and year of publication
Elevation	Shuttle Radar Topography Mission (SRTM)	30 m		[33]
Slope	SRTM	30 m		[34]
Aspect—sines	SRTM	30 m		[34]
Aspect—cosines	SRTM	30 m		[34]
Flow accumulation	SRTM	30 m		[35]
Distance to marshlands	World Agroforestry Centre (ICRAF)	Shapefile		[36]
Distance to river networks	SRTM	30 m		[37]
Distance to open water bodies	Landviewer, Sentinel 2 L2A	10 m		[35]
Topographic Position Index (TPI)	SRTM	30 m		[34]
Topographic Wetness Index (TWI)	SRTM	30 m		[34]
Normalised Difference Water Index (NDWI)	Landviewer, Sentinel 2 L2A	10 m		[35]
Normalised Difference Vegetation Index (NDVI)	Landviewer, Sentinel 2 L2A	10 m	27th of February 2019	[38]
			27th of July 2019	
			15th of September 2019	
Population density	Worldpop	100 m		[35]

### Data analysis

All collected citizen science data were aggregated in Microsoft Excel, and included a unique code for each of the volunteers, as well as their location (latitude and longitude of the house of the volunteer), the collection date, the mosquito species (indoors and outdoors) and its feeding status, the presence/absence of confirmed malaria cases in the 2 weeks prior to data collection, and the perceived mosquito nuisance expressed on a five-point Likert scale [from ‘no nuisance’ (0) to ‘very much nuisance’ (5)].

ArcGIS pro 2.4 (ESRI, Redlands, CA) was used to compile geographic data and create maps of the study area. Locations of the selected households were used to make an interpolation of mosquito abundance using the inverse distance weighting (IDW) method [39].

Means, and proportions of confirmed malaria cases or perceived mosquito nuisance reported as well as mosquito species compositions were calculated. Spearman correlation coefficients were calculated using Statistical Package for the Social Sciences (Version 25.0, IBM Corporation, New York, USA) to determine the relationships among perceived mosquito nuisance, confirmed malaria cases reported and the number of mosquitoes (Culicidae) or number of *An. gambiae* s.l. collected (Fig. 2). For all analyses, the results from the indoor and outdoor traps were summed per household. Calculations of these correlations were made at different levels of aggregation: with raw data for individual households, resulting in approximately 1344 data points (i.e. 112 households × 12 months), or with village averages, resulting in 60 data points (i.e. 5 villages × 12 months). In addition, to investigate the variability/consistency of correlations among villages, correlations were calculated separately for each village, using the raw data at household level.

Environmental factors (Table 1) were selected to evaluate their impact on the abundance of mosquitoes (Culicidae) and *An. gambiae* s.l. as collected via the citizen science approach in the study area. To do so, Pearson correlations and multiple linear regressions were conducted to explore the relationship between mosquito abundance and selected environmental variables. The results were analysed at the sector level (all sampling points in the Ruhuha area). For the multiple regression analyses, the *leaps* package in R3.5 was used, which employs an iterative process for finding one or more ‘best subsets’ of the explanatory variables [40]. To visually inspect cross-correlations among the thirteen variables, principal component analysis (PCA) was used. Finally, negative binomial GLMs (*MASS* package in R) were run to evaluate models that included or excluded ‘village’ as additional variable to account for spatial clustering of the observations.

## Results

Overall, 112 volunteers participated in the current study by reporting perceived mosquito nuisance and confirmed malaria cases, and by submitting mosquitoes for a period of 12 months. In total, 51% of volunteers were male, and 49% were female. The median age of the group was 42 years (minimum 24 years, maximum 68 years). Participation varied from village to village, and included 12 volunteers from Busasamana, 35 from Kagasera, 24 from Kibaza, 10 from Kiyovu, and 35 from Mubano (Fig. 1). Data collected by two volunteers who moved away from the selected villages during the study were excluded in the analyses.

### Perceived mosquito nuisance

**Fig. 3:**
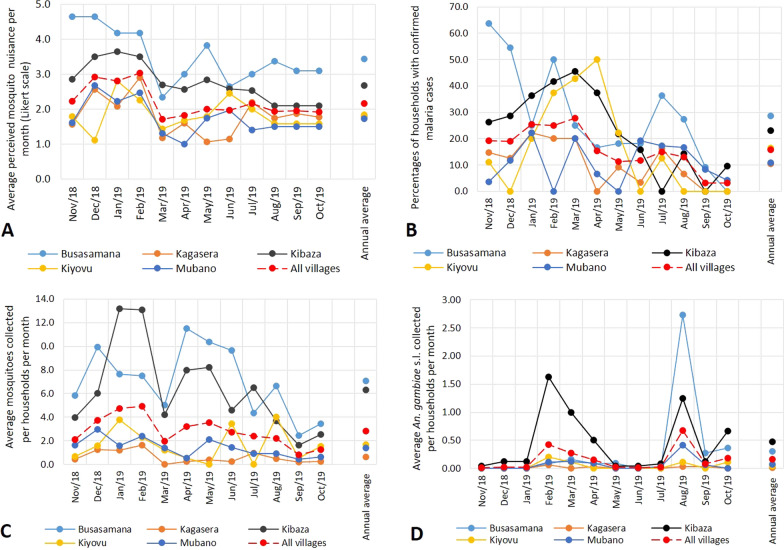
**A** Spatial and temporal distribution of variables determined. **A** average perceived mosquito nuisance, **B** proportion of households reporting at least one confirmed malaria case, **C** average number of mosquitoes (all species) per month, and **D** average number of *An. gambiae* s.l. per month reported in the five selected villages of Ruhuha sector, Rwanda

At village level, there was a clear spatial and temporal variation in the perception of mosquito nuisance. The highest average (± standard deviation) perceived mosquito nuisance for the whole period was reported from Busasamana (3.4 ± 0.5), followed by Kibaza (2.7 ± 0.1), both in the south of the Ruhuha sector. Volunteers from Kagasera and Kiyovu in the eastern part of the sector experienced little nuisance (1.8 ± 0.4) followed by volunteers from Mubano in the north (1.8 ± 0.2). At a temporal scale, the highest average mosquito nuisance scores were reported in December (2.9 ± 1.3), January (3.0 ± 0.9), and February (3.1 ± 0.8) 2019 (Fig. 3A).

### Confirmed malaria cases

Over the entire 1-year study period, every month on average 16% of the volunteers reported having at least one confirmed malaria case in the two weeks prior to sampling. However, 66% of the households reported at least one confirmed malaria case in their household throughout the study period. The highest average monthly percentage of households over the entire year having a confirmed malaria case was reported in Busasamana (28.7%), followed by Kibaza (23.1%), Kiyovu (16.3%), Mubano (14%), and Kagasera (10%). Over the year of the study, the month with the highest percentage of households reporting having at least one confirmed malaria case was March (27.7%), followed by January (25.5%) and February (25%). The months with the lowest percentage of households having a confirmed malaria case were September and October 2019 with 3.2% each (Fig. 3B).

### Mosquito species composition and molecular identification of members of the *An. gambiae* complex

A total of 3793 female mosquitoes were collected in the five selected villages using a handmade carbon dioxide baited trap over a period of 1 year. Of these, 51.7% (n = 1964) were collected indoors and 48.2% (n = 1829) were collected outdoors. These mosquitoes belonged to four genera and 10 species were identified. Of all mosquitoes, 89.4% (n = 3390) was morphologically identified as culicine and 10.6% (n = 403) as anopheline (Table 2). Among female anopheline mosquitoes collected, 90.8% (n = 366) were unfed and 9.2% (n = 37) were fed. Of the *Anopheles* species, 49.6% (n = 200) were collected indoors and 50.4% (n = 203) were collected outdoors. Of the total culicines, 76.6% (n = 2905) were *Culex* species, with *Culex quinquefasciatus* (74%) as the most abundant *Culex* species, followed by *Mansonia* (11.4%) and *Coquillettidia* species (1.3%).

**Table 2:** Species composition of mosquitoes collected during the citizen science programme in five villages in Ruhuha sector, Rwanda, November 2018–October 2019

Village name	Busasamana	Kagasera	Kibaza	Kiyovu	Mubano	Total	% Species composition
*An. gambiae* s.l.	42	5	135	5	28	215	5.7
*An. maculipalpis*	0	0	5	0	3	8	0.2
*An. pharoensis*	4	0	12	0	1	17	0.4
*An. squamosus*	0	0	0	0	1	1	0.0
*An. ziemanni*	26	1	131	2	2	162	4.3
Total *Anopheles* spp.	72	6	283	7	35	403	10.6
*Coquillettidia* spp.	15	16	15	2	3	51	1.3
*Culex* spp.	732	213	1329	155	476	2905	76.6
*Mansonia* spp.	156	14	183	22	59	434	11.4
Total Culicinae	903	243	1527	179	538	3390	89.4
Total Culicidae	975	249	1810	186	573	3793	100
% *Anopheles* spp.	17.9	1.5	70.2	1.7	8.7		
% Culicidae	25.7	6.6	47.7	4.9	15.1		

Of the *Anopheles* species, 53.3% were *An. gambiae* s.l. (n = 215), 40.2% were *Anopheles ziemanni*, and the other species were *Anopheles pharoensis* (4.2%, n = 17), *Anopheles maculipalpis* (2%; n = 8) and *Anopheles squamosus* (0.2%; n = 1) (Table 2). Volunteers in Kibaza collected the highest proportion of *Anopheles* species (70.2%), followed by Busasamana (17.9%), Mubano (8.7%), Kiyovu (1.7%) and Kagasera (1.5%) (Table 2). Kibaza had the highest proportion of *An. gambiae* s.l. (33.5%), followed by Busasamana (10.4%), Mubano (6.9%), Kiyovu (1.2%) and Kagasera (1.2%).

**Fig. 4:**
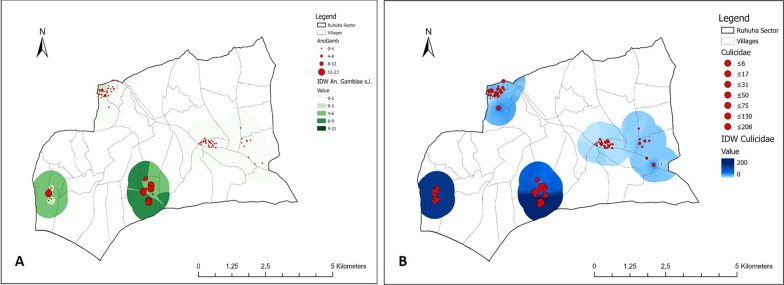
Maps of Culicidae (**B**) and *An. gambiae* s.l. (**A**) collected in the Ruhuha, Rwanda. Intensity of colouring represents estimated abundance based on inverse distance weighting as interpolation method

At sector level, relatively more mosquitoes were collected in the south of Ruhuha sector than in the north. Especially in Busasamana and Kibaza, the villages that also reported the highest nuisance and malaria levels, more Culicidae and *An. gambiae* s.l. were collected in comparison with the three other villages (Fig. 4).

At sector level, the average (± standard deviation) number of mosquitoes (Culicidae) collected per household per month was 2.8 (± 1.2). Busasamana had the highest average number of mosquitoes (7.1 ± 6.3) per household per month, followed by Kibaza (6.3 ± 9.3). The lowest catch of mosquitoes was recorded in Kagasera (0.6 ± 1.6) followed by Mubano (1.4 ± 2.7) and Kiyovu (1.6 ± 3.0) (Fig. 3C). For *An. gambiae* s.l., volunteers from Kibaza had the highest average of *An. gambiae* s.l. (0.47 ± 1.5) per household per month followed by Busasamana (0.30 ± 1.6), Mubano (0.07 ± 0.4), Kiyovu (0.04 ± 0.2) and Kagasera (0.01 ± 0.1) (Fig. 3D). At a temporal scale, most mosquitoes (Culicidae) were caught in January and February 2019 while the lowest numbers were caught in September and October 2019 (Fig. 3C). Both the months February and August 2019 had a peak in the number of *An. gambiae* s.l. in comparison with other months (Fig. 3D), and the number of *An. gambiae* s.l. dropped from March to May.

#### Sporozoite rates and molecular identification of members of the *An. gambiae* complex

The *P. falciparum* sporozoite infection rate for all 403 female *Anopheles* was 0%. Of the 20% (42 out of 215) of *An. gambiae* s.l. tested, 62% (26/42) were *Anopheles arabiensis*, 31% (13/42) were *An. gambiae* sensu stricto and 7% (3/42) did not yield a PCR product.

#### Correlation between number of mosquitoes collected and mosquito nuisance reported

**Fig. 5:**
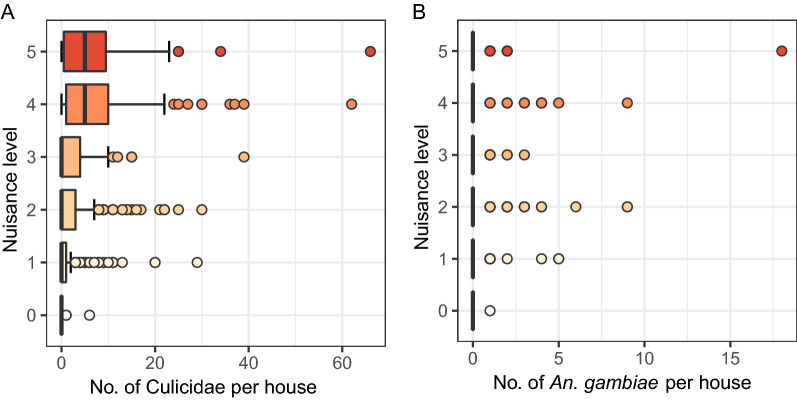
Boxplots showing correlation between perceived mosquito nuisance and total mosquitoes collected. The correlation between perceived mosquito nuisance experienced per household per month with the total number of mosquitoes (Culicidae) (**A**) or *An. gambiae* s.l. (**B**) collected per household per month in five selected villages in Ruhuha sector, Rwanda is shown

Based on all data from the entire sampling period, there was a moderate, positive correlation between perceived mosquito nuisance reported per household per month and the number of mosquitoes (Culicidae) per household per month (r_s_ = 0.459; *P* < 0.0001; Fig. 5A) and a weak, positive correlation between nuisance and number of *An. gambiae* s.l. per household per month (r_s_ = 0.121; *P* < 0.0001; Fig. 5B). It should be noted that, in case of *An. gambiae* s.l. collections, only 7.2% of the collections contained one or more individuals in the trap (Fig. 5B), whereas this proportion was 42.5% in case of total Culicidae.

**Fig. 6:**
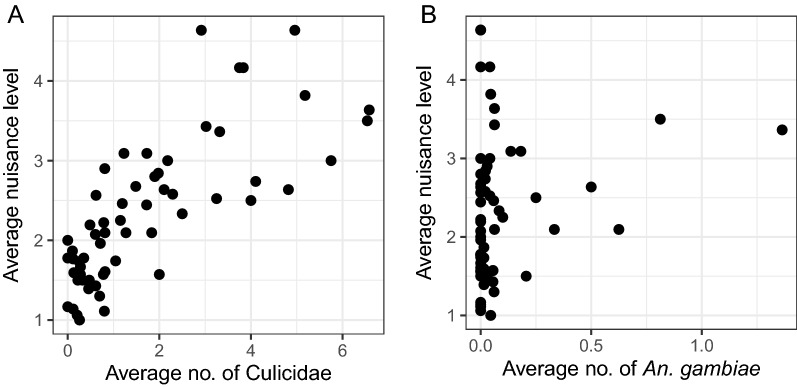
Scatter plots showing the correlation between perceived mosquito nuisance and number of mosquitoes. The correlation is between average perceived mosquito nuisance per village per month and average number of mosquitoes (Culicidae, **A**) and *An. gambiae* s.l. (**B**) per village per month reported by the volunteers in five selected villages in Ruhuha sector, Rwanda

Interestingly, when the same data were aggregated and averaged by village, there was a strong correlation between the average nuisance level and average number of mosquitoes per month per village (r_s_ = 0.798; *P* < 0.0001; Fig. 6A). In other words, the average perceived mosquito nuisance level could be explained by the average number of mosquitoes collected. However, there was no significant correlation between the average nuisance level and the number of *An. gambiae* s.l. (r_s_ = 0.225; *P* = 0.084; Fig. 6B).

When correlations were investigated for each village separately, there were significant correlations between the level of perceived mosquito nuisance and the total number of mosquitoes (Culicidae) collected per household per month, except for Busasamana where no significant correlation was found. Similar to the analyses for the entire sector, no significant correlations were observed between mosquito nuisance and the number of *An. gambiae* s.l. collected (Table 3).

**Table 3:** Spearman correlation coefficients for the relationship between nuisance level and the total number of Culicidae/*An. gambiae* s.l. separately for each village

	Mosquito group	r_s_	*P*
Busasamana	Culicidae	0.117	0.175
	*An. gambiae*	0.044	0.613
Kagasera	Culicidae	**0.266**	**< 0.001**
	*An. gambiae*	0.046	0.385
Kibaza	Culicidae	**0.371**	**< 0.001**
	*An. gambiae*	0.056	0.374
Kiyovu	Culicidae	**0.392**	**< 0.001**
	*An. gambiae*	0.057	0.583
Mubano	Culicidae	**0.413**	**< 0.001**
	*An. gambiae*	0.037	0.507

In bold the significant correlations are highlighted

#### Correlation between perception of mosquito nuisance and confirmed malaria cases

**Fig. 7:**
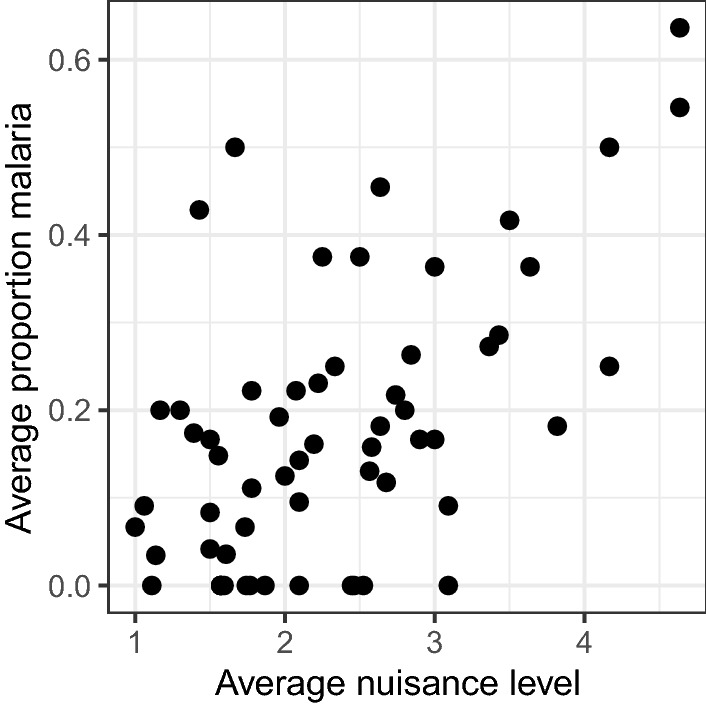
Correlation between average mosquito nuisance and average proportion of confirmed malaria cases. Scatterplot showing the correlation between average mosquito nuisance level and average proportion of confirmed malaria cases reported per village per month by the volunteers in five selected villages in Ruhuha sector, Rwanda

When data from households were aggregated and averaged for each village, a moderate, positive correlation between perceived mosquito nuisance and proportion confirmed malaria cases per households per month (r_s_ = 0.473, P < 0.001) was found (Fig. 7). When this correlation was investigated for each village separately, there was a significant, strong correlation for Kibaza (r_s_ = 0.643, *P* = 0.023), while for Busasamana (r_s_ = 0.567, *P* = 0.054), Kagasera (r_s_ = 0.261, *P* = 0.413), Kiyovu (r_s_ = 0.223, *P* = 0.486), and Mubano (r_s_ = − 0.138, *P* = 0.670), correlations were not significant.

#### Correlation between mosquitoes collected and confirmed malaria cases reported

**Fig. 8:**
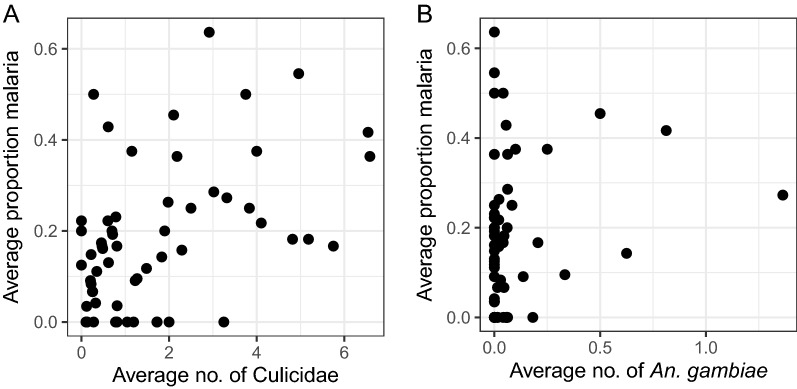
Correlation between average proportion of malaria in households and number of mosquitoes. Scatter plots showing the correlation between average proportion of malaria in households per village per month and average number of mosquitoes (Culicidae) (**A**) and *An. gambiae* s.l. (**B**) per village per month reported by the volunteers in five selected villages in Ruhuha sector, Rwanda

At village level, a moderate, significant correlation (r = 0.468, *P* < 0.0001) was found between the average number of mosquitoes and the proportion of confirmed malaria cases reported per village per month (Fig. 8A). No correlation was found between the average number of *An. gambiae* s.l. collected and the proportion of confirmed malaria cases reported per village per month (r = 0.204, *P* = 0.124; Fig. 8B). When data were analysed separately by village, no significant correlations were found between the number of mosquitoes or *An. gambiae* s.l. and the presence of confirmed malaria cases reported per village per month, except for Kibaza where a correlation (r = 0.581, *P* = 0.047) was found between number of Culicidae and confirmed malaria cases (Table 4).

**Table 4:** Pearson correlation coefficients between number of Culicidae/*An. gambiae* s.l. and presence of confirmed malaria cases in five villages of Ruhuha sector, Rwanda

Village name	Mosquito group	r	*P*
Busasamana	Culicidae	0.170	0.597
	*An. gambiae*	− 0.126	0.696
Kagasera	Culicidae	0.560	0.058
	*An. gambiae*	− 0.183	0.569
Kibaza	Culicidae	**0.581**	**0.047**
	*An. gambiae*	0.394	0.205
Kiyovu	Culicidae	− 0.241	0.450
	*An. gambiae*	0.252	0.430
Mubano	Culicidae	− 0.154	0.632
	*An. gambiae*	0.174	0.590

Significant correlations are highlighted in bold

#### Environmental risk factors explaining the spatial distribution of mosquitoes and malaria vectors

**Fig. 9:**
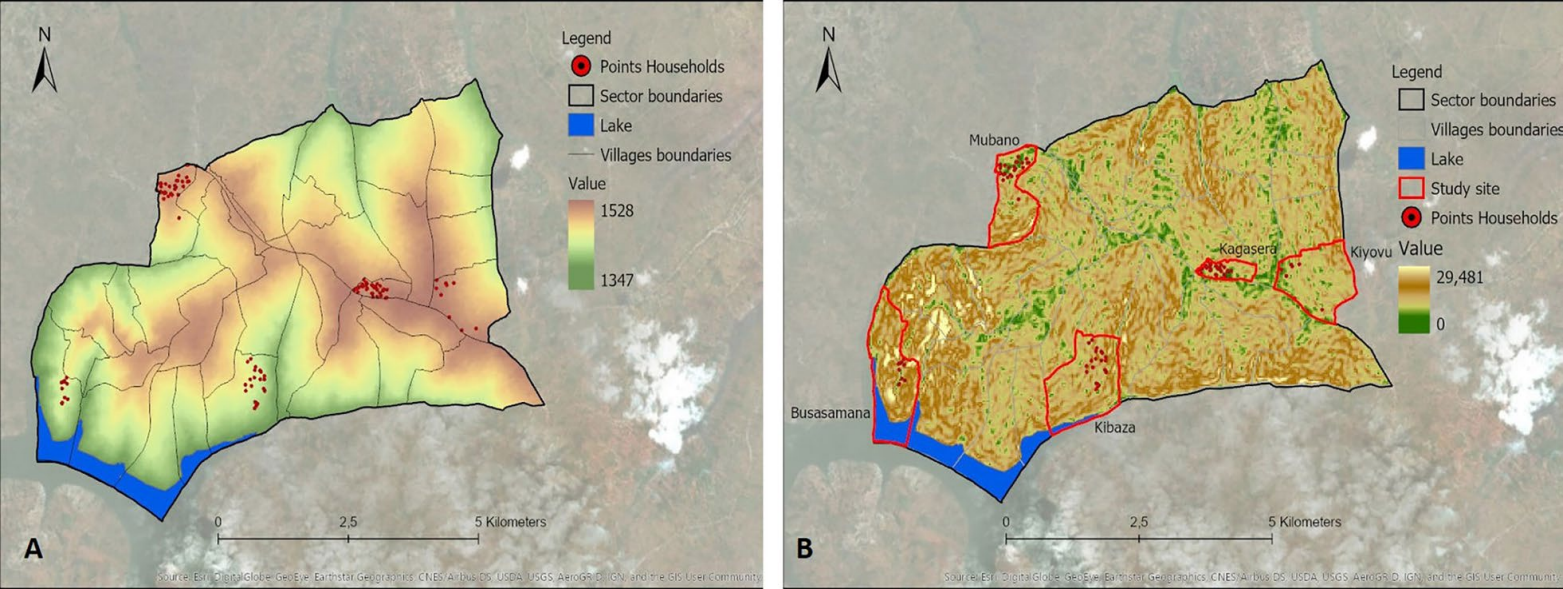
Maps showing elevation (**A**), slope (**B**)

**Fig. 10:**
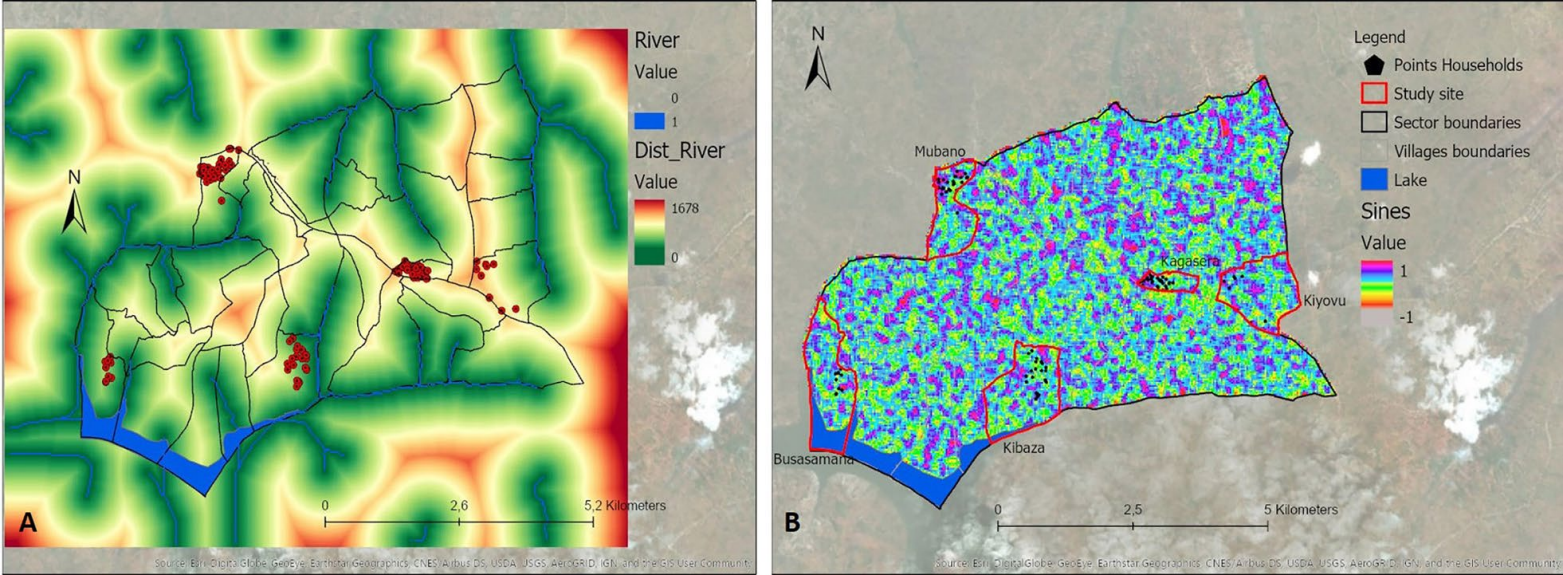
Maps showing distance to river (**A**), sines aspect (**B**)

**Fig. 11:**
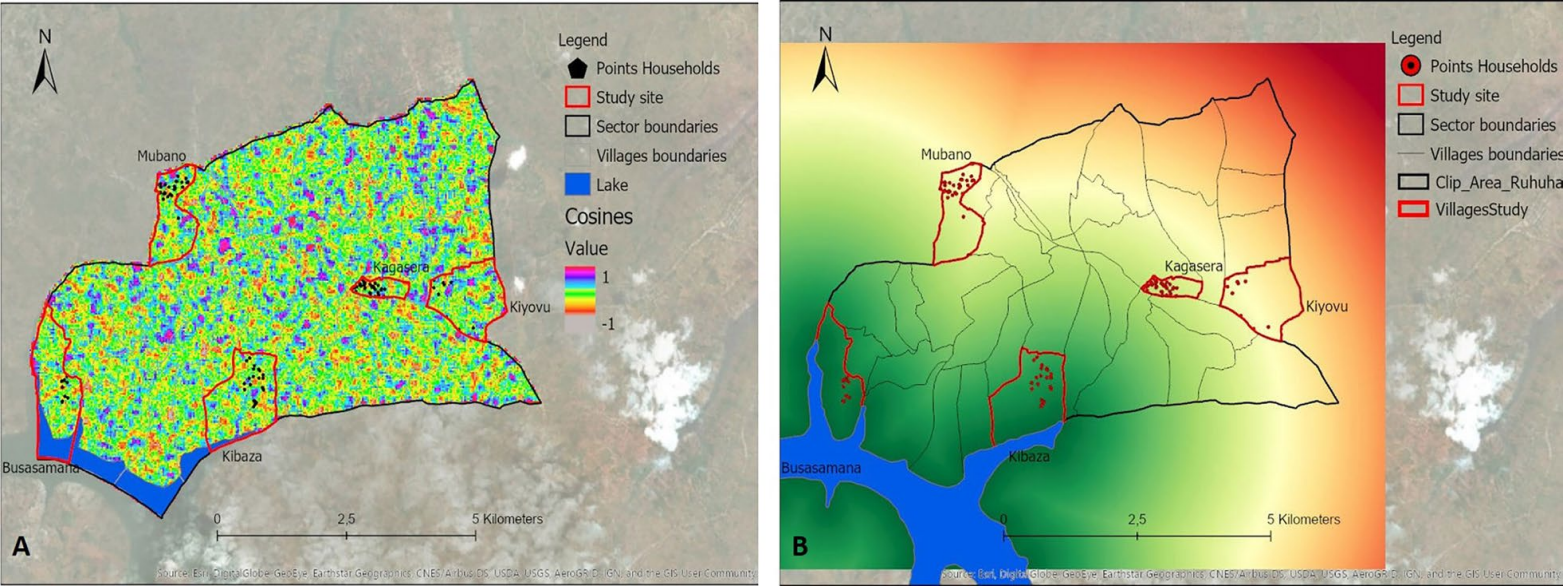
Maps showing cosines aspect (**A**), distance to lakes (**B**)

**Fig. 12:**
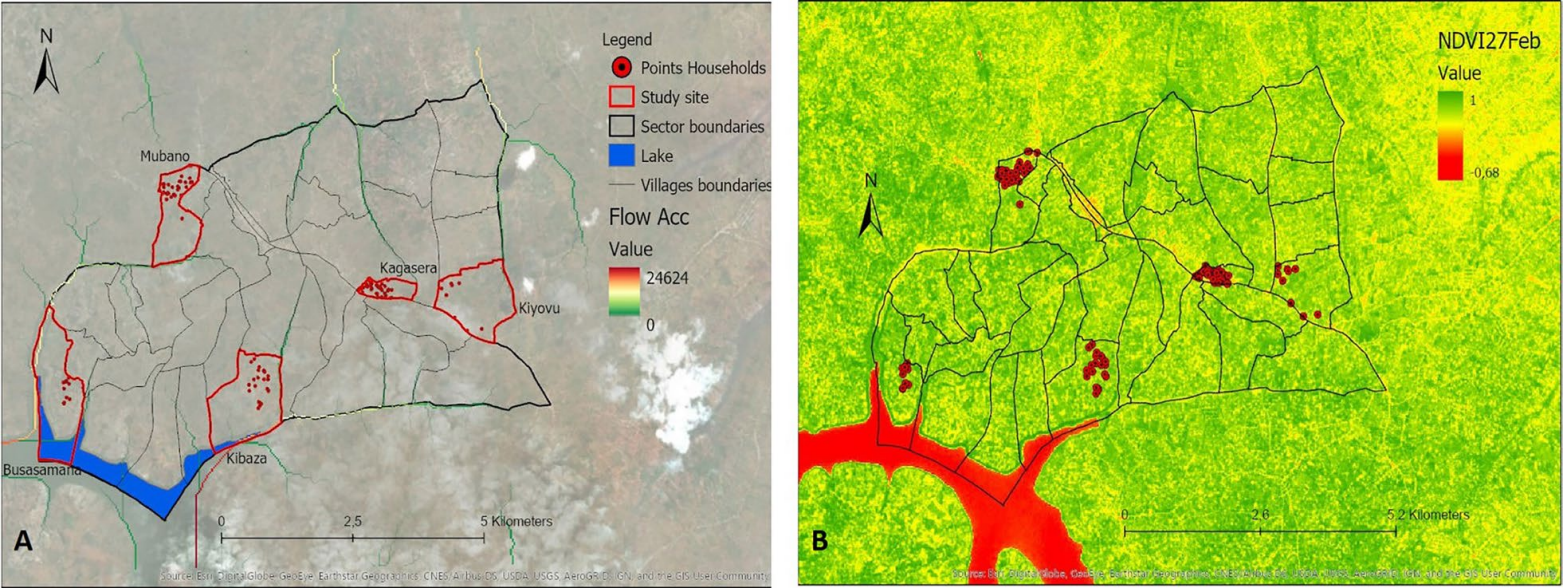
Maps showing flow accumulation (**A**), Normalized Difference Vegetation Index (NDVI) of the study area on the 27th of February 2019 (**B**)

**Fig. 13:**
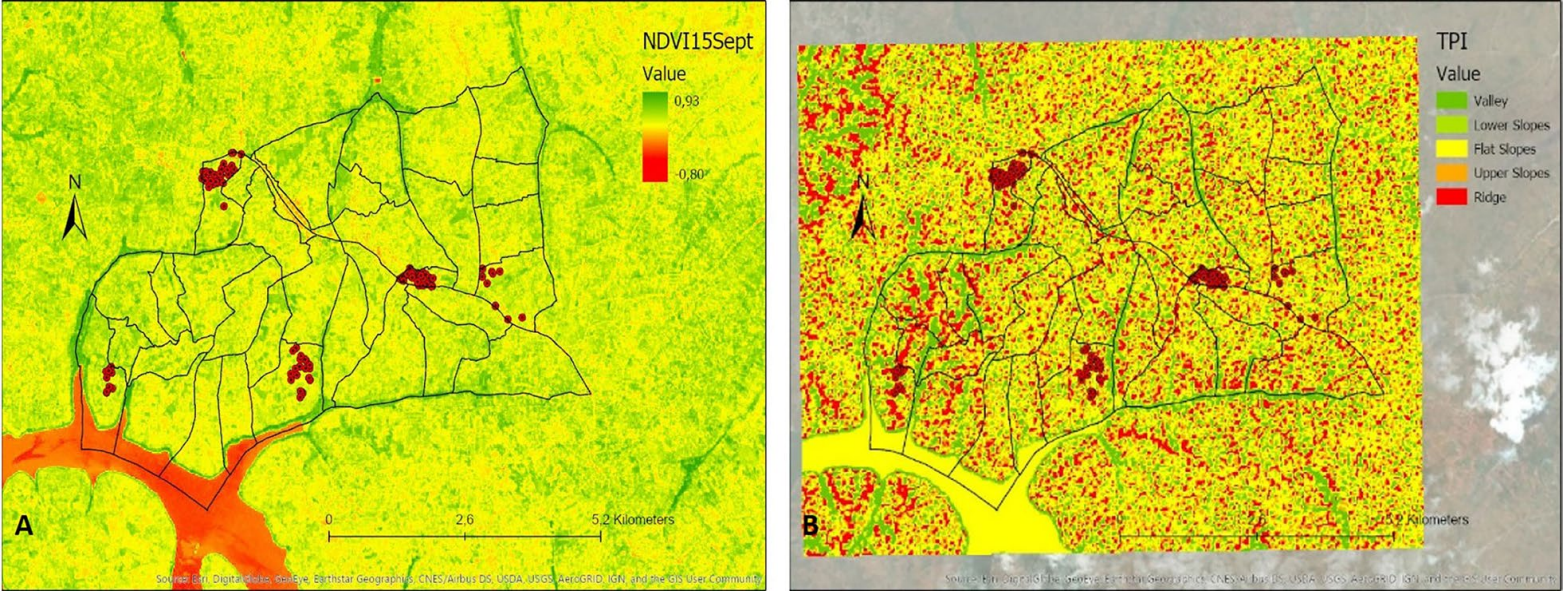
Maps showing Normalized Difference Vegetation Index (NDVI) of the study area on the 27th of July 2019 (**A**), NDVI of the study area on the 15th September 2019 (**B**)

**Fig. 14:**
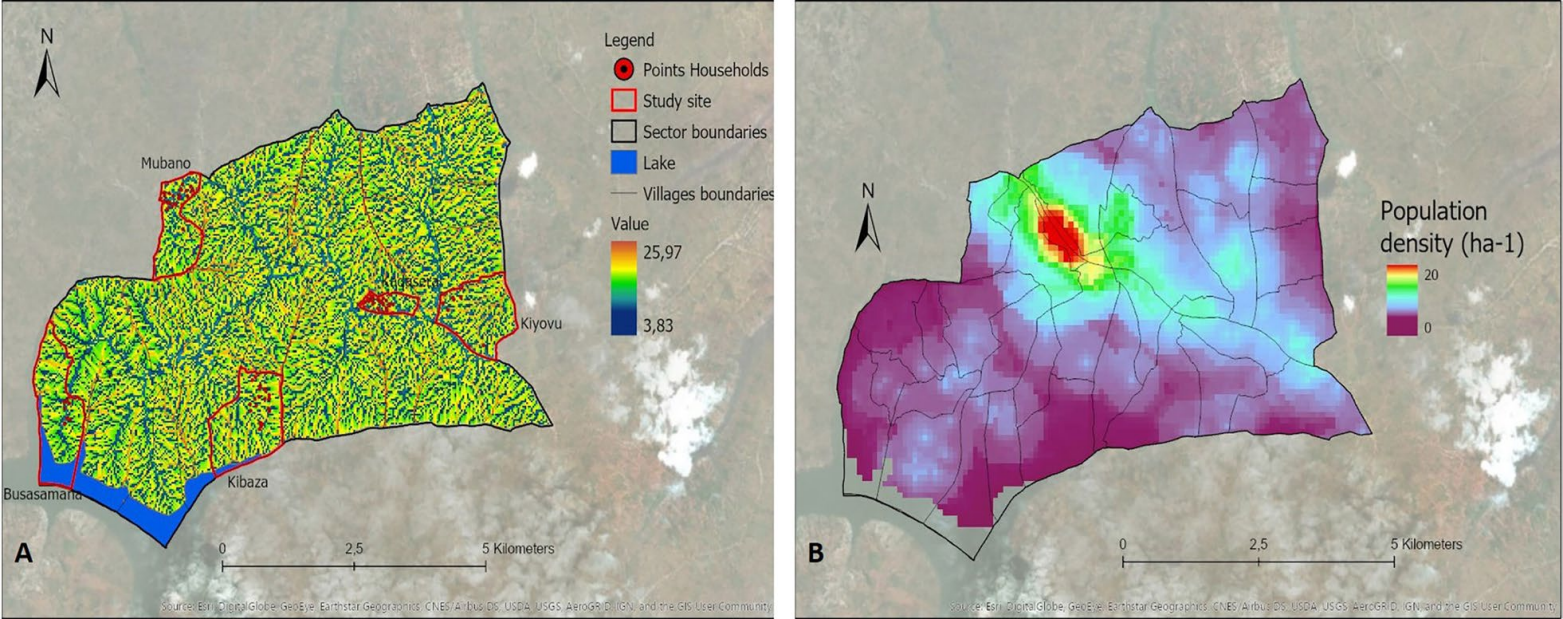
Maps showing Topographic Wetness Index (TWI) (**A**), population density (**B**)

Thirteen variables identified from the literature were selected (Table 1). These included 12 environmental variables: elevation, slope, distance to marshlands, distance to open water, distance to the river network, flow accumulation, cosines of the aspect, sines of the aspect, Normalized Difference Vegetation Index (NDVI), Normalized Difference Water Index (NDWI), Topographic Wetness Index (TWI), Topographic Position Index (TPI), as well as one demographic variable, population density. Data for these variables were extracted from different data sources (Table 1). Values for the different variables were derived and calculated from the extracted data specific to the area under study (Ruhuha sector; Figs. 9, 10, 11, 12, 13 and 14) and linked to the locations of and data from the households under study.

Investigation of the bivariate correlations between the selected environmental variables and the abundance of mosquitoes (Culicidae and *An. gambiae* s.l.) revealed that there were significant, negative correlations between distance to marshland, distance to open water, distance to rivers, NDVI and population density for both mosquito groups (Table 5). For Culicidae, also the sines of aspect, slope and TPI showed a significant correlation.

**Table 5:** Pearson correlation coefficients between mosquito group and environmental factors (sector scale)

Variables	Culicidae			*An. gambiae* s.l.		
	r	R^2^	P	r	R^2^	P
Distance to marsh	− 0.51**	0.25	< 0.01	− 0.40**	0.16	< 0.01
Distance to water	− 0.67**	0.48	< 0.01	− 0.45**	0.2	< 0.01
Distance to river	− 0.56**	0.30	< 0.01	− 0.42**	0.17	< 0.01
Elevation	− 0.69**	0.47	< 0.01	− 0.49**	0.24	< 0.01
Flow accumulation	− 0.10	0.01	0.31	− 0.02	0.00	0.81
Cosines of aspect	− 0.15	0.02	0.12	− 0.13	0.02	0.19
Sines of aspect	− 0.21*	0.05	0.03	− 0.15	0.02	0.12
NDVI	0.29**	0.09	< 0.01	0.20*	0.04	0.04
NDWI	− 0.11	0.01	0.26	− 0.03	0.00	0.73
Slope	0.26**	0.07	0.01	0.18	0.03	0.06
TPI	0.20*	0.04	0.03	0.10	0.01	0.28
TWI	− 0.14	0.02	0.15	− 0.09	0.01	0.38
Population density	− 0.66**	0.44	< 0.01	− 0.48*	0.23	< 0.01

*P<0.05. **P < 0.01

**Fig. 15:**
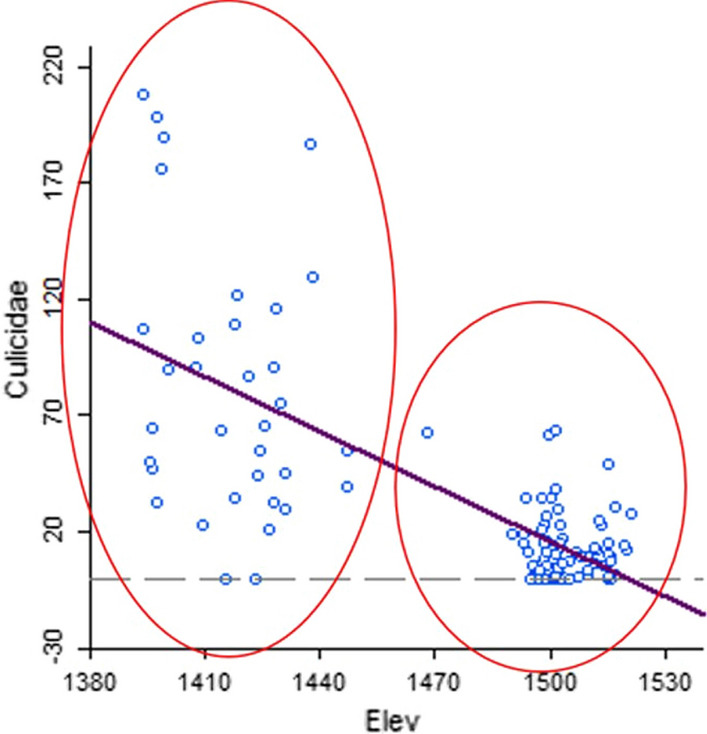
Scatterplot showing correlation between total Culicidae and elevation (in metres)

At sector level, there was a relationship between elevation and the total number of mosquitoes (Table 5) and in this case there was a clear distinction between the south and the north of the area. In Fig. 15, the right oval encompasses data points from the villages in the north (Kagasera, Mubano and Kiyovu), whereas the left oval includes data points from the two villages in the south (Kibaza and Busasamana).

**Fig. 16:**
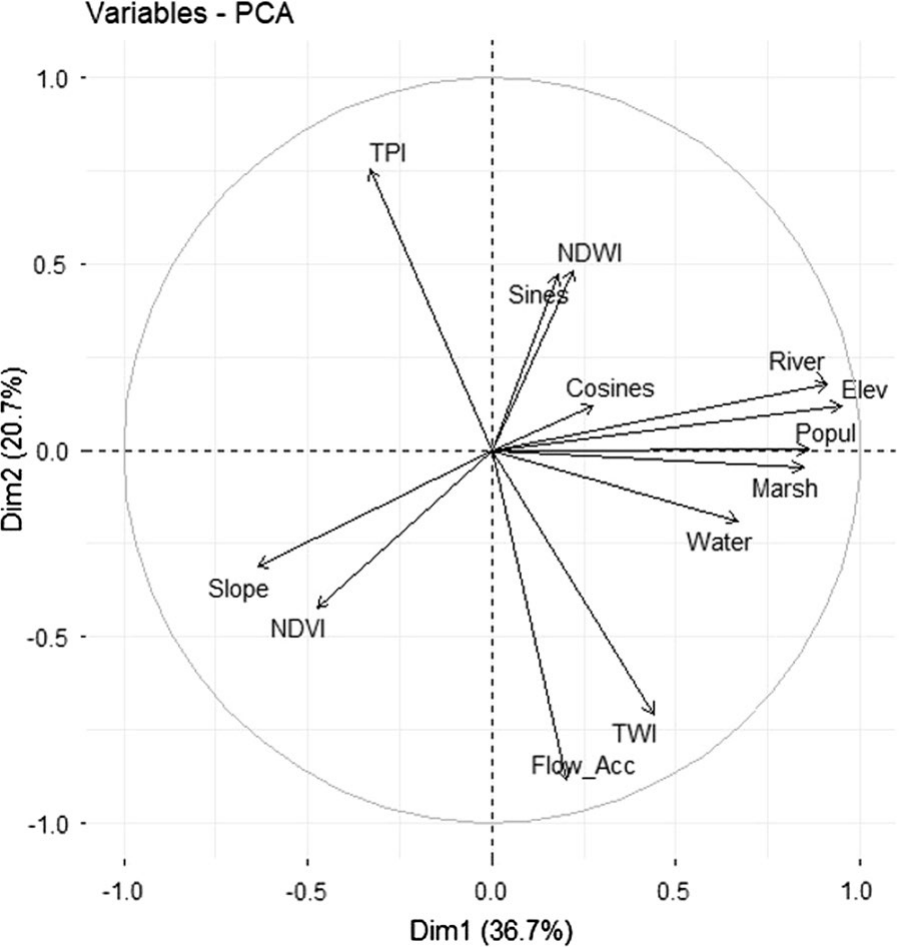
Biplot of the selected environmental variables. The variables include elevation (Elev), slope, distance to marshlands (Marsh), distance to open water (Water), distance to the river network (River), flow accumulation (Flow_Acc), cosines of the aspect (Cosines), sines of the aspect (Sines), Normalized Difference Vegetation Index (NDVI), Normalized Difference Water Index (NDWI), Topographic Wetness Index (TWI), Topographic Position Index (TPI), as well as one demographic variable, population density (Popul)

Prior to performing the multiple regression analysis, correlations among the thirteen selected variables were visually inspected by means of principal component analysis. This showed that NDWI and the sines of the aspect are highly correlated, as the direction and length of their vectors are similar. This also applies for correlations among the variables distance to river, distance to marshlands, elevation and population. On the other hand, the variables NDVI and NDWI showed a negative correlation as they diverge and form a large angle (Fig. 16).

**Fig. 17:**
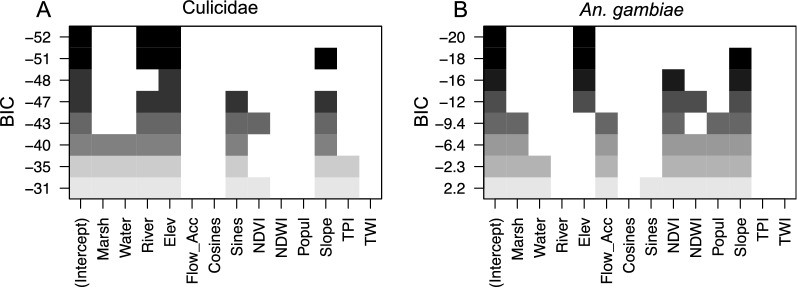
Multiple linear regression models showing selection of best subsets based on *leaps* package in R. Filled squares indicate the inclusion of a variable in a model. Performance of a model increases from the bottom to the top of each panel based on Bayesian Information Criterion (BIC in **A** and **B**)

Multiple regression analyses, followed by ‘best subset’ model selection in the *leaps* package of R, revealed that when using the Bayesian Information Criterion (BIC), only distance to the river network and elevation remained in the model (Fig. 17A). For the *An. gambiae* s.l. model, using BIC, only elevation remained in the final model (Fig. 17B). Elevation and distance to river thus seem to have a role in explaining (malaria) mosquito abundance, despite the high correlation among the two variables (Fig. 16). This could be interpreted as follows: the larger the distance is from the river network the lower the risk to encounter mosquitoes or collecting them, and the higher the elevation (where the population in Ruhuha is mostly concentrated), the lower the risk to encounter mosquitoes including *An. gambiae* s.l.

Based on the *leaps* variable selection, final negative binomial models were run that also included ‘village’ as a variable next to elevation and distance to the river network. This was then compared to the model that excluded village. In this way, possible spatial variation in the data caused by differences among villages was accounted for. Consistent with the analyses in *leaps*, the model without village showed that, for *An. gambiae*, elevation is a significant predictor (*β* = − 0.033, Z = − 3.39, *P* < 0.001), and distance to the river network is not (*β* = 186.0, Z = 0.95, *P* = 0.34). The addition of village to the model did not improve its performance, as a comparison among both models (with and without village) was not significant (Likelihood Ratio statistic = 8.97, df = 4, *P* = 0.062). Similarly, for Culicidae, elevation was a significant predictor (*β* = − 0.030, Z = − 4.27, *P* < 0.001), but in contrast to the *leaps* analyses, distance to the river network was not significant in this model (*β* = 231.4, Z = 1.61, *P* = 0.11). The addition of village did not improve model performance, because there was no significant difference between the model that included and the model that excluded village (Likelihood Ratio statistic = 7.02, df = 4, *P* = 0.13). Based on these outcomes, it was concluded that there was no variation in Culicidae or *An. gambiae* that could be explained by village.

## Discussion

The potential of employing mosquito collection tools with the aid of citizens was investigated in this study. In combination with data on perception of mosquito nuisance denoted in a questionnaire, data from these collection tools can be used as a proxy for areas with higher risk of malaria. More specifically, the findings from this study provide insights into the spatial and temporal dynamics of mosquitoes in five selected villages of Ruhuha based on citizen science data. This was further assessed for *An. gambiae* s.l. separately, as this is the most important malaria vector in the area [41]. Furthermore, the study highlighted environmental risk factors that explain these spatial dynamics indicating areas with higher risk of malaria, especially in the south of the study area, although parasite infection rate was zero for the *An. gambiae* s.l. collected during the study.

All the anopheline species found in the current study were also reported from other parts of Rwanda [5]. *Anopheles gambiae* s.l. was the most abundant of all anopheline species and was recorded every month in all five villages. However, populations were not highly abundant throughout the season. This can be partially explained by the fact that the handmade trap has a lower trapping efficiency in comparison to other trapping technologies at household level. Most mosquitoes (Culicidae) were caught in the months of January and February 2019 during the short rainy season, but most malaria vector mosquitoes were collected in August 2019 (long dry season). Although larval habitats in this period have dried out, this is also the period in which the second cycle of rice cultivation starts, leaving the irrigated fields with little vegetation, but with sufficient water for mosquito breeding, thereby increasing adult abundance. The villages of Busasamana and Kibaza (Fig. 1), both located in the south or Ruhuha, are good examples in this regard, because the peak in *An. gambiae* s.l. was especially observed here. Busasamana has two irrigated fields, namely Nyaburiba, a rice field, and Nyagafunzo, used for irrigated cultivation of subsistence crops [42]. Kibaza has also one irrigated rice field nearby (known under the same name, i.e. Kibaza). *Anopheles gambiae* s.l. is generally associated with irrigated rice, and irrigation elevates relative humidity which enhances survival of these vectors [43]. Although no *P. falciparum* infection was found in the *An. gambiae* s.l. collected in the studied area, the citizen science approach was able to identify areas with a relatively higher malaria vector abundance which are at higher risk for malaria as evidenced by the highest self-reported proportion of malaria cases in these two villages.

Similar to these results, a study from Ethiopia found that larval and adult abundance of the malaria vectors *An. arabiensis* and *An. pharoensis*, was higher in a village with nearby irrigation than in a village without nearby irrigation, as was malaria prevalence [44]. Another study conducted in Malawi showed that changes in the geography of breeding potential across irrigated spaces can have profound effects on the distribution of malaria risk for those living in close proximity to irrigated agricultural schemes [45, 46].

In the present study, Kagasera had the lowest number of mosquitoes including *An. gambiae* s.l. The reason could be that the village is characterized by a higher quality of houses and that the village is located further away from the water network, which is not the case for Busasamana and Kibaza. A study conducted in rural The Gambia demonstrated that incorporating a ceiling made from locally available materials significantly reduced house entry by *An. gambiae* [47]. Another study conducted in The Gambia demonstrated that there were lower vector survival rates and less malaria in villages with a higher proportion of metal roofs. The indoor climate of metal-roof houses, characterized by lower humidity and higher temperatures may reduce the survival of indoor-resting mosquitoes and may have even contributed to the observed reduction of malaria in parts of sub-Saharan Africa [48]. Another reason could be that Kagasera has a higher elevation compared to Busasamana (see Fig. 9A) and this could also have had an impact on the presence of *An. gambiae* s.l. A study conducted in Mambilla Plateau, Northeast Nigeria, demonstrated that indeed, altitude can influence mosquitoes and *Anopheles* species abundance [49].

Findings on the role of environmental factors showed that in particular elevation and distance to the river network contributed to the spatial distribution in numbers of mosquitoes and *An. gambiae* s.l. A study conducted in Mara River basin located in the southwestern part of Kenya and the north-eastern side of Tanzania demonstrated that distance to nearby human habitation was another important factor influencing mosquito larval abundance. Most of the breeding habitats were recorded within a distance of 70–450 m from the nearest human habitation [50]. In the same study, it was found that in the river habitats, more mosquitoes were found in slow flowing streams and riverbeds with little vegetation as compared to open water, an indication that low-lying wet land with grassy vegetation such as marshlands may play an important role in harbouring malaria vectors [50].

It is concluded that perceived mosquito nuisance can be used as an indicator for mosquito density. However, although significant correlations between mosquito nuisance and the number of mosquitoes and *An. gambiae* s.l. were found when data were aggregated for all 12 months of the study, the correlations with *An. gambiae* s.l. were absent when analysed separately for each village or when using village level averages. In other words, nuisance seemed to be strongly driven by the total numbers of mosquitoes, and not by the abundance of *An. gambiae* s.l. Interestingly, in an earlier cross-sectional study conducted in 2017 and 2018, a moderate and significant correlation with nuisance was found for both mosquitoes and *An. gambiae* s.l. One of the reasons of this difference could be that the total number of mosquitoes for both years was almost three times higher (9965) and almost two times higher for *An. gambiae* s.l. (974) than in the present study. Possibly, to detect correlations between mosquito numbers and nuisance a minimum number of mosquitoes needs to be collected.

**Fig. 18:**
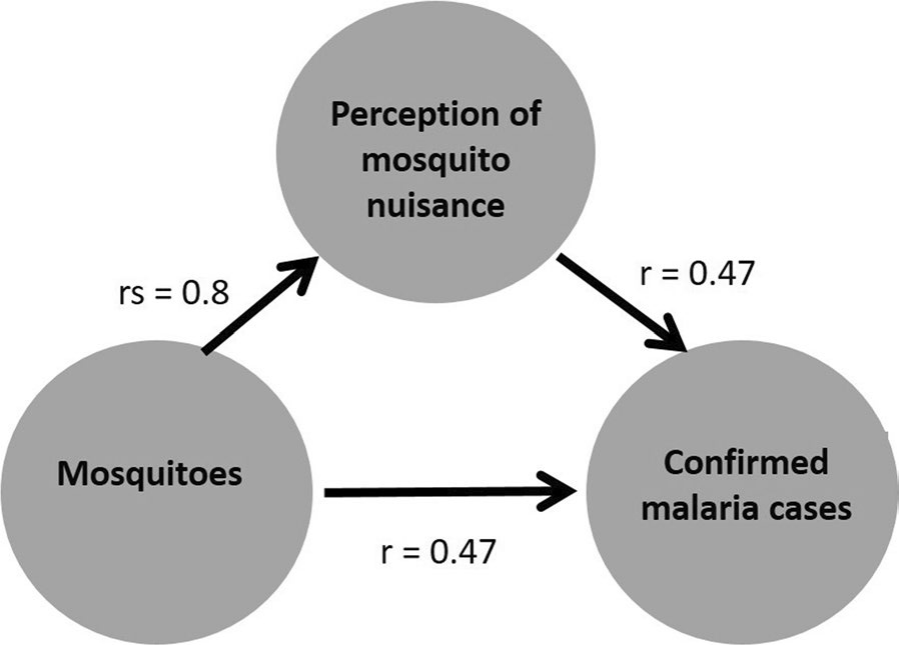
Strength of correlations of mosquito nuisance, mosquitoes (Culicidae) and malaria cases. Strength of correlations between mosquito nuisance (Culicidae) and proportion of malaria cases, and between number of mosquitoes (Culicidae) collected and malaria cases

Interestingly, significant correlations of similar strength between mosquito nuisance and proportion of malaria cases, and between number of mosquitoes collected and malaria cases (both r = 0.47) were shown (Fig. 18). At the start, the study aimed to investigate whether nuisance level can be used as an indicator for malaria risk. Results suggest that, in current study, nuisance is an equally strong indicator for malaria risk as the number of mosquitoes collected. Although a correlation between numbers of *An. gambiae* s.l. and malaria cases was expected to be found, the number of *Anopheles* individuals was low compared to, for example, what was collected during the baseline survey and, therefore, correlations were probably absent when using the citizen science data for this one species separately.

For the calculation of the correlations reported in this study, it would have been preferable to have all 112 volunteers more homogeneously distributed over the sector, but this was not feasible for logistical reasons, and hence citizens in five village clusters were worked with. The model analyses showed that elevation was the most important factor explaining mosquito abundance, and that village as a factor did not add significant explanatory power. It is interesting to note that the proportion of confirmed malaria cases reported per village per month was correlated (r = 0.468, *P* < 0.0001) (Fig. 8A) with the average number of Culicidae collected, even though 89% of these mosquitoes were non-malaria vectors.

Busasamana and Kibaza both located in the south of the study area, had the highest number of *An. gambiae* s.l., mosquito nuisance and the highest percentage of households having a confirmed malaria case indicating that the highest intensity of malaria transmission in the study area strongly relates to land use and altitude (Figs. 9 and 10A). A same proportion of host-seeking *An. gambiae* s.l. was collected both indoors and outdoors suggesting that transmission can take place both indoors and outside the house.

## Conclusions

The results demonstrate that a well-established citizen science network provides valuable information on the bionomics of (malaria vector) mosquito species. In combination with reports on perceived mosquito nuisance, the citizen science network provides indications on the spatial and temporal variation in the risk of malaria. The study shows that especially elevation and distance to the river network explained the spatial variation of (malaria vector) mosquitoes at the sector level. An option to consider for Rwanda is the expansion of the current surveillance network of 12 sentinel sites with a citizen science network to areas where no monitoring is established. Data collected through a citizen science programme may be similarly useful for the planning of malaria vector control strategies by public health departments in other African countries. In this way, such a citizen science network could eventually contribute to more effective spending of limited resources for vector control.

## Data Availability

The datasets used and analysed in this manuscript are available from the Digital Archive Network Services (DANS-EASY) repository at 10.17026/dns-xhq-jtfs.

## Abbreviations

BIC: Bayesian Information Criterion
CSP: Circumsporozoite protein
DNA: Deoxyribonucleic acid
DEM: Digital Elevation Model
ELISA: Enzyme-linked immunosorbent assay
HMIS: Health Management Information System
IDW: Inverse distance weighting
IRS: Indoor residual spraying
ITN: Insecticide-treated bed nets
MOPDD: Malaria and Other Parasitic Disease Division
NDVI: Normalised Difference Vegetation Index
NDWI: Normalised Difference Water Index
PCA: Principal component analysis
PCR: Polymerase chain reaction
SRTM: Shuttle Radar Topography Mission
SPSS: Statistical Package for the Social Sciences
UV: Ultraviolet
TPI: Topographic Position Index
TWI: Topographic Wetness Index
